# Delayed Diagnosis of a Bilateral Congenital Diaphragmatic Hernia in an Infant: A Rare Case Presentation

**DOI:** 10.7759/cureus.77968

**Published:** 2025-01-25

**Authors:** Fatema Alameen, Rashed Almusalam, Meaad Alansari, Abdulrahman Alshafei

**Affiliations:** 1 Surgery, Bahrain Defence Force Royal Medical Services, Riffa, BHR; 2 Anaesthesia, Bahrain Defence Force Royal Medical Services, Riffa, BHR; 3 Pediatric Surgery, Bahrain Defence Force Royal Medical Services, Riffa, BHR

**Keywords:** bilateral diaphragmatic hernia, bochdalek hernia, congential diaphragmatic hernia, diaphragmatic hernia repair, late presenting cdh, morgagni diaphragmatic hernia, pediatric surgery outcomes, rare pediatric presentations

## Abstract

We report the case of a four-month-old male infant diagnosed incidentally with bilateral congenital diaphragmatic hernias. Our patient was found to have chest asymmetry during an unrelated hospital visit and bilateral diaphragmatic defects were confirmed on cross-sectional imaging. Surgical repair of a right-sided Bochdalek hernia and a left-sided Morgagni hernia was performed with excellent outcomes. This case report documents the rare occurrence of a bilateral late-presenting congenital diaphragmatic hernia, augments the limited existing knowledge, and highlights the variability in clinical outcomes. It provides valuable perspectives on the potentially improved survival rates in the uncommon manifestations of this condition.

## Introduction

A congenital diaphragmatic hernia (CDH) occurs when a defect in the diaphragm allows intra-abdominal organs to herniate into the thorax [1]. The majority of cases are diagnosed antenatally; however, some present postnatally, with presentations ranging from mild respiratory distress to cyanosis, depending on the size of the defect and the degree of pulmonary impairment [1]. Bilateral CDH is rare (2%) and is associated with high mortality [2]. 

In 2017, a cohort study of 80 patients with bilateral CDH by Botden et al. found that the survival rate of these patients was as low as 26%. It was associated with larger defects and smaller lungs [2]. In 2003, Neville et al. reported a low survival rate (35%) in an analysis of 17 patients with bilateral CDH. This was attributed to the association between bilateral CDH and major anomalies [3].

In this paper, we report a rare case of late-presenting bilateral CDH that was surgically repaired with excellent results.

## Case presentation

Clinical presentation

We report the case of a four-month-old male infant, who was brought to the emergency department due to an accidental overdose of simethicone. On examination, the vital signs were as follows: body temperature, 36.9 °C; blood pressure, 90/60 mmHg; heart rate, 127 beats per minute; respiratory rate, 30 breaths per minute; and oxygen saturation, 99% on room air. The patient weighed approximately six kilograms. Chest examination revealed equal air entry with no added sounds and normal heart sounds. However, his chest was asymmetrical, with a slight protrusion of the left anterior chest wall. On further questioning, the mother reported that this had been present for a month. She denied feeding difficulties or respiratory symptoms. The patient was born via spontaneous vaginal delivery to a 20-year-old healthy mother. He had a normal physical examination at birth with an uneventful hospital stay and was discharged on day two of life.

**Figure 1:**
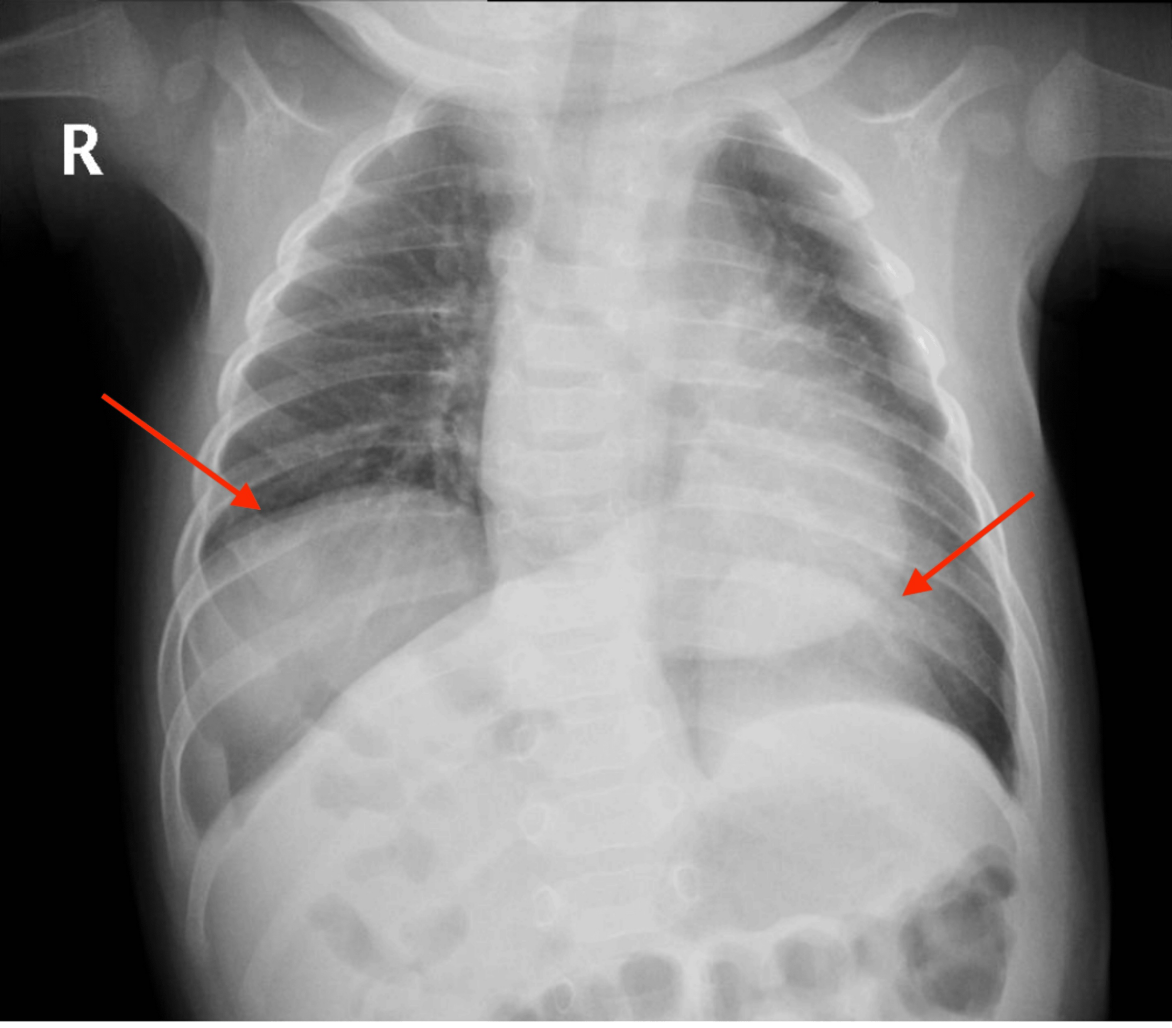
Chest radiograph showing the bilateral diaphragmatic hernia (red arrows)

A chest radiograph (chest X-ray) was suggestive of a congenital diaphragmatic hernia (Figure 1).

**Figure 2:**
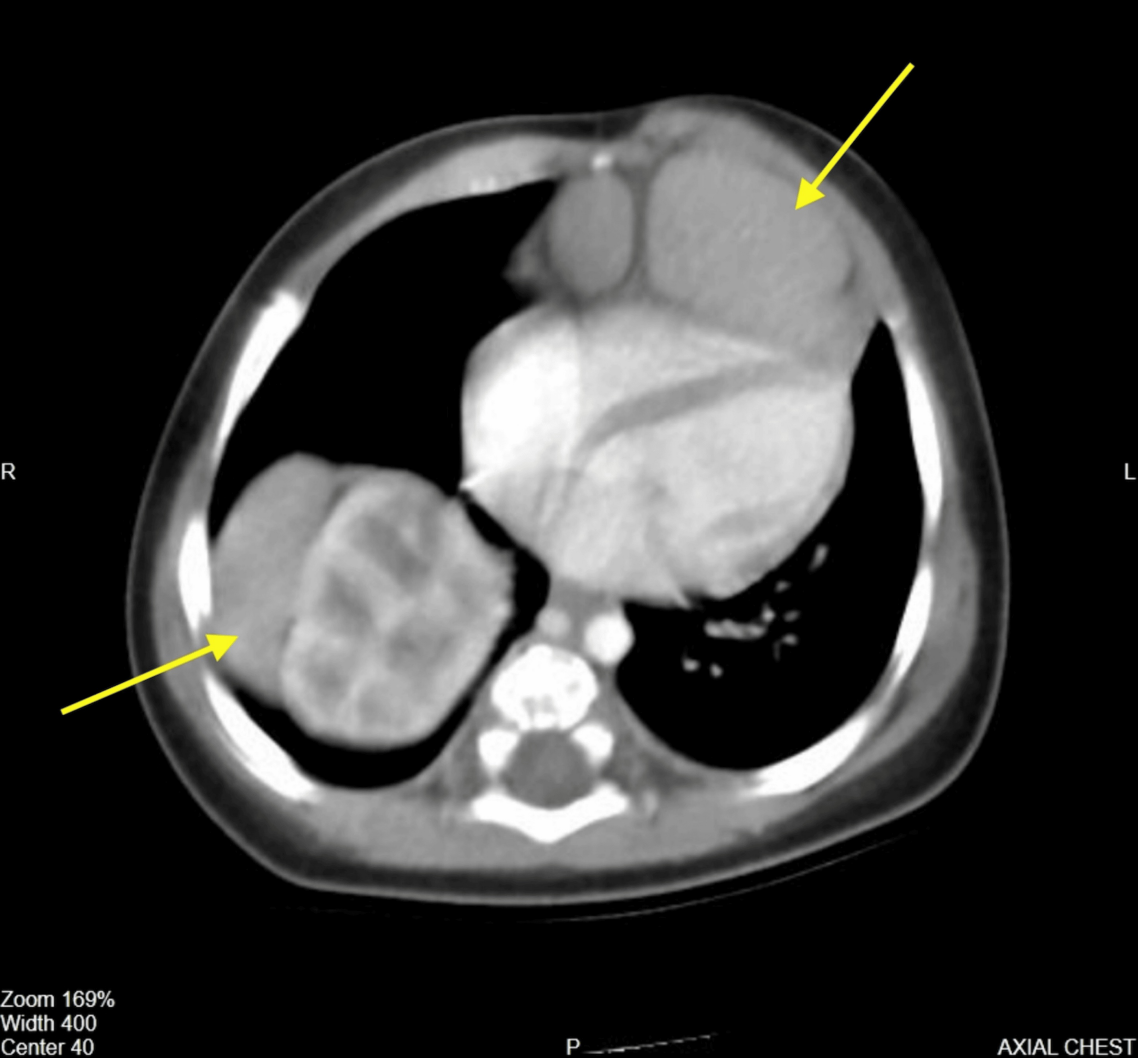
Axial view of the chest CT showing abdominal organs herniated into the thoracic cavity (yellow arrows)

An abdominal and chest computed tomography (CT) revealed that the right kidney and a part of the right lobe of the liver extended into the thoracic cavity on the right posterolateral side. A part of the left lobe of the liver was also seen extending into the thoracic cavity, lying behind and to the left of the sternum and anterior to the heart, causing a protrusion in the left chest wall (Figure 2).

Otherwise, the bilateral lung parenchyma and abdominal solid organs appeared normal. The pediatric trans-esophageal echocardiography showed a three millimeter patent foramen ovale with a left to right shunt.

Surgical management

The patient underwent diagnostic laparoscopy, which showed a left Morgagni retrosternal hernia containing the left lobe of the liver with a sac adherent to the pericardium. A right Bochdalek posterolateral hernia was also visualized containing the right lobe of the liver, right kidney, adrenal gland, and colon. After confirming the diagnosis, an upper midline transverse incision was made to repair the defects. 

The contents on the right side were reduced with difficulty as the liver and kidney were adherent to the pleura and the chest wall. The right CDH sac was excised, and two-layer primary closure was performed using 2-0 Prolene (PROLENE™ 2-0, Ethicon, New Jersey, US) and 3-0 Prolene (Surgipro™ II 3-0, Covidien, Dublin, Ireland) interrupted sutures. A Morgagni defect was identified, containing the left lobe of the liver. The hernial sac was adherent to the pericardium and was left in place. Primary repair was performed with interrupted horizontal U-sutures using 2-0 Prolene between the posterior rim of the diaphragm and the anterior abdominal wall. On-table chest radiography confirmed successful repair with mild right-sided pneumothorax.

Postoperative course

**Figure 3:**
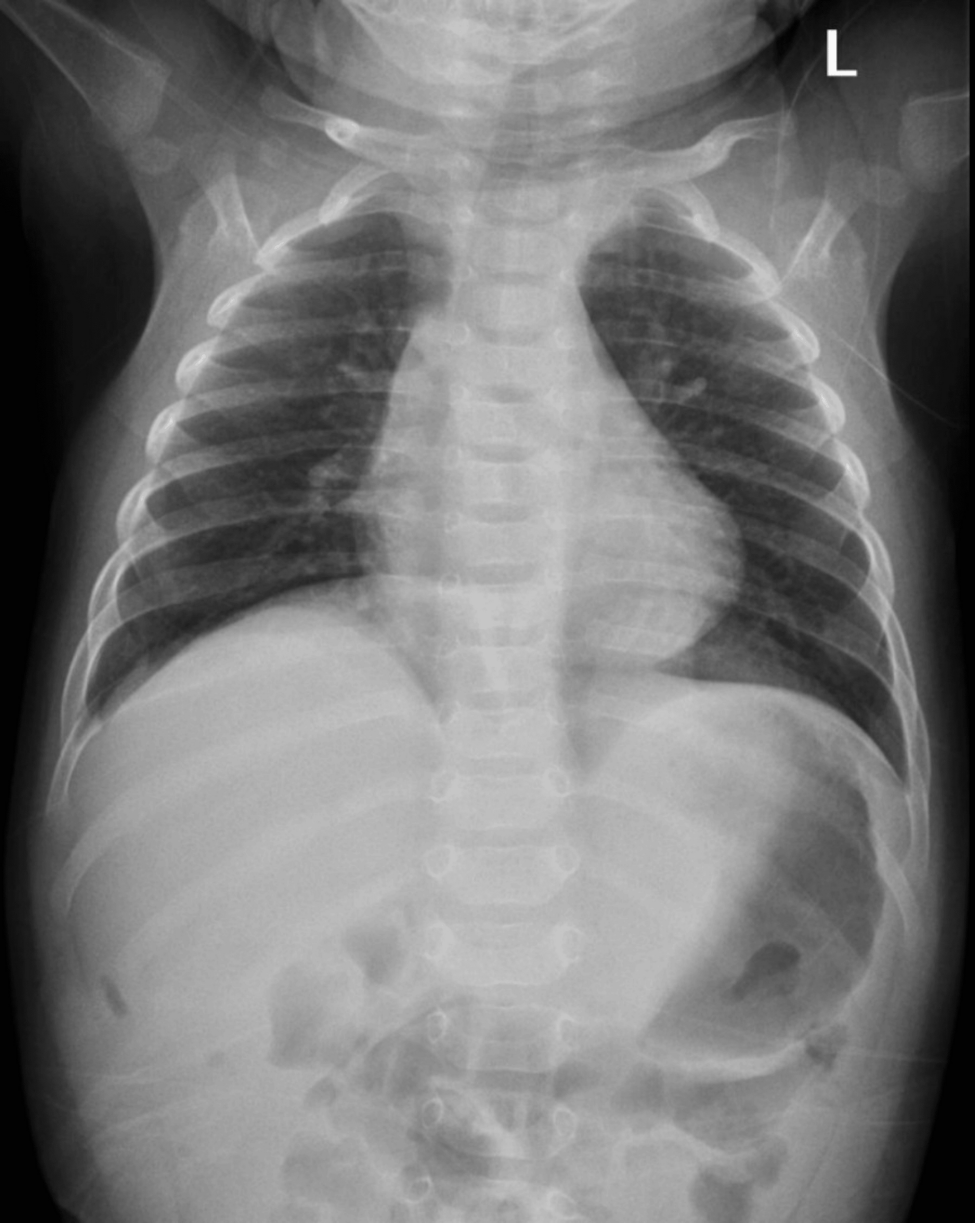
Normal chest radiograph on the seventh day after the surgery

The patient was extubated in the operating theatre and transferred to the intensive care unit for 24-hour observation. Feeds were started the following day and were tolerated well by the patient. A repeat chest radiograph showed a resolution of the right pneumothorax (Figure 3) and the patient was discharged on the seventh day after the surgery.

At present, the patient is one year and nine months old. The most recent follow-up evaluation occurred six months prior, during which the patient exhibited no symptoms and demonstrated age-appropriate developmental milestones.

## Discussion

The diaphragm normally develops at 12 weeks of gestation from the septum transversum and pleuroperitoneal membranes [1]. Disruption of this process results in CDH [1]. To date, the mortality and morbidity rates of CDH remain high and are directly related to the degree of pulmonary hypoplasia and hypertension associated with the defect. Babies are usually born with severe respiratory distress requiring immediate intubation and meticulous hemodynamic support to ensure the maintenance of systemic perfusion and lower pulmonary pressure before definitive repair. 

Bilateral CDH is rare and typically has an unfavorable prognosis owing to severe pulmonary hypertension and hypoplasia [1,2]. In a retrospective analysis of patients born with bilateral CDH between 1995 and 2015, the mortality rate was 74% [2]. In contrast, late-presenting CDH is in itself a reflection of improved lung development and, therefore, favorable survival outcomes. One study reported 100% survival for late-presenting CDH outside the neonatal period [4]. It may present with various clinical signs. Patients may exhibit either acute or chronic respiratory or gastrointestinal symptoms or may be asymptomatic [4]. Our patient did not experience any respiratory compromise, apart from the unusual chest wall asymmetry due to the anterior liver herniation on the left side.

In general, liver herniation remains a poor prognostic factor, especially in large right-sided Bochdalek defects [5]. Our patient, however, did not experience any respiratory compromise despite a right-sided liver herniation. In some instances, the liver may prevent the bowel and stomach from herniating into the right thorax, allowing for normal fetal lung development [5]. This phenomenon is more likely to occur when the diaphragmatic defect is small and likely accounts for the normal lung development in our patient. In addition, the echogenic profile of the liver can resemble that of lung tissue on antenatal ultrasound scans, potentially leading to a missed or late diagnosis of CDH. 

This case combines two rare entities of CDH, bilateral and late-presenting, making it an extremely rare occurrence. The incidence of these two entities occurring concomitantly is difficult to determine as our literature review mostly yielded case reports. Upon reviewing studies of late-presenting CDH, we found that bilateral defects were uncommon in this group. In a study that included 79 patients with late-presenting CDH, only one case had bilateral defects (1.2%) [4]. A literature review on late-presenting CDH found that out of the 362 patients included in the study, only four patients had bilateral defects (1.1%) [6].

## Conclusions

This case highlights the unique combination of two rare forms of CDH, bilateral and late-presenting. While bilateral CDH typically has a poor prognosis due to severe pulmonary hypoplasia and hypertension, our patient exhibited a remarkably favorable outcome with no significant respiratory compromise despite the presence of right-sided liver herniation. Given the rarity of bilateral late-presenting CDH, this case contributes to the limited literature and emphasizes the variability in clinical outcomes, offering insights into the potential for improved survival in certain atypical presentations.
